# Gender differences in quality of life in adults with long-standing type 1 diabetes mellitus

**DOI:** 10.1186/s13098-020-00571-x

**Published:** 2020-07-17

**Authors:** Ana María Castellano-Guerrero, Raquel Guerrero, Desireé Ruiz-Aranda, Sofia Perea, Alfonso Pumar, Federico Relimpio, Miguel Angel Mangas, Fernando Losada, Maria Asunción Martínez-Brocca

**Affiliations:** 1 Servicio de Endocrinología y Nutrición, Hospital Universitario Virgen Macarena, Avenida Dr Fedriani 3, 41009 Seville, Spain; 2 Servicio de Endocrinología y Nutrición, Hospital Universitario Virgen del Rocío, Seville, Spain; 3 Instituto de Biomedicina de Sevilla (IBiS), Hospital Universitario Virgen del Rocío/Consejo Superior de Investigaciones Científicas/Universidad de Sevilla, Seville, Spain; 4 Psicología, Universidad Loyola Andalucía, Seville, Spain; 5 Dpt. of Genetics, Beth Israel Deaconess Medical Center, Harvard Medical School, Boston, USA

**Keywords:** Type 1 diabetes mellitus, Quality of life, Gender differences

## Abstract

**Background:**

To assess gender differences in Quality of life (QoL) and in sociodemographic, clinical and psychological factors associated with impaired QoL in adults with long-standing type 1 diabetes mellitus (DM1).

**Methods:**

Cross-sectional evaluation in a random cohort of DM1 adult patients from a tertiary care hospital. QoL was evaluated using translated and validated self-administered Diabetes QoL questionnaire (Es-DQoL), and results transformed into a 0–100 scale. Psychological assessment included a planned psychological interview and self-reported questionnaires (Beck Depression Inventory II, State-Trait Anxiety Inventory Form Y, Fear of hypoglycaemia Scale, Medical Outcomes Study Social Support Survey).

**Results:**

A total of 312 patients (51.6% male; 38.2 ± 12.7 years; HbA_1c_ 7.5 ± 1.1% (58.5 ± 14.2 mmol/mol); 20.4 ± 12.0 years of DM1) were included in the analysis. Male and female subgroups showed similar sociodemographic and diabetes-related features and comparable social support. Among female patients, higher frequency of depression [31.7% (IC95% 26.2–40.8) vs. 14.9% (IC95% 10.1–20.8), p < 0.05] and anxiety [23.2% (IC95% 19.3–33.14) vs. 13.0% (IC95% 8.1–18.4), p < 0.05] and severity of depressive and anxious symptoms were also found. Compared to male patients, female patients showed lower QoL [75 (IC95% 73.6–77.5) vs. 80 (IC95% 75.7–83.1), p < 0.05] and scored significantly worse in subscale Diabetes-related worries [69 (IC95% 50.0–81.0) vs. 75 (IC95% 72.9–79.0), p < 0.05]. Fear of hypoglycemia and severity of depressive and anxious symptoms were factors independently associated to lower QoL in men and women while high frequency of glycemic excursions was a female-specific predictive one.

**Conclusions:**

Adult women with long-standing DM1 showed lower QoL probably related to higher frequency and severity of psychopathological syndromes. Depressive and anxious symptoms and, among women, exposure to glycemic excursions were identified as modifiable, QoL-related variables. Educational, technological and psychological interventions are needed in order to improve QoL in DM1 patients.

## Background

A major goal of diabetes care is to prevent acute and late diabetes complications. Consistent data suggest that maintaining glucose levels as close as possible to the normal range prevents or delays diabetic complications [1, 2]. Intensified insulin regimen through both multiple daily insulin injections and continuous subcutaneous insulin infusion have been proven effective in achieving near-euglycemia in type 1 diabetes (DM1) [1, 3]. Living with DM1 encompasses adequate knowledge and skills, appropriate interpretation of frequent self-monitoring blood glucose levels, management of complex insulin regimen, awareness of diabetes complications and a constant self-care, a challenging process with a potential negative impact on quality of life (QoL). As intensified insulin therapy is linked to a threefold increase in frequency of severe hypoglycemia, implementation of intensive treatment has increased the appearance of fear of hypoglycemia [1]. The impact of hypoglycemia as a predictor of morbidity and mortality has generated an increasing interest in the last years. Moreover, fear of hypoglycemia has a relevant psychological impact and while controlling for clinical factors, could be an important diabetes-specific determinant of health-related usefulness, a generic metric of health status [4].

Behavior, psychological adjustment, depression, anxiety, parenting, family environment, glycemic control and micro- and macrovascular disease are potential factors influencing QoL in diabetic population [5]. The influence of gender on QoL among diabetic population and specifically among DM1 patients is not well-established as previous reports included mainly children and adolescents, young adults or adults on continuous subcutaneous insulin infusion therapy [6–11]. In addition, lack of consensus definitions and guidelines has led to the use of QOL measures that are often imprecise and inappropriate [12]. Evaluation of diabetes-specific variables associated with QoL in cohorts of adult DM1 patients has been reported [13–16]. However, general and diabetes-specific psychopathological features such as fear for hypoglycemia are only included in a limited number of studies [15, 16].

In this cross-sectional study, the primary objective was to assess gender differences in QoL in DM1 adult patients; the secondary objective was to identify gender-related factors affecting QoL.

## Methods

### Study design

Cross-sectional evaluation carried out at the Diabetes Unit of University Hospital Virgen del Rocío (UHVR). UHVR is a tertiary care DM1 reference center, the largest complex of the Andalusian public healthcare system, serving a population of 554.981 inhabitants. At the time of this evaluation, 1665 DM1 adolescent and adult patients were included in the UHVR Registry. Internal Review Committee at UHVR approved the study protocol. All patients signed informed consent prior to inclusion in the study.

### Patients’ selection

Adult patients (ages 18–65 years) with DM1 [according to American Diabetes Association criteria (ADA 2014) [17] for at least 1 year, were randomly selected at UHVR using a computer-generated random list. Exclusion criteria included pregnancy or pregnancy planning; psychiatric or neurological disorders limiting their ability to complete the questionnaires and any non-diabetes related clinical condition that could affect the evaluation. Patients selected were informed about the aims of the study by their physician and if agreed to participate, they were contacted by phone (for up to three times) to schedule an appointment.

### Quality of life evaluation

QoL was assessed by the self-administered Diabetes QoL (DQoL) questionnaire translated and validated into Spanish (Es-DQoL). The DQOL questionnaire showed high reliability as well as internal and external validity, both for the original version and the Spanish translation [18]. It contains 43 items that patients rank on 5-point Likert scale ranging from 1 to 5 (1 = never; 5 = all the time). Four subscales measure diabetes impact on daily life (17 items, range: 17–85), diabetes-related worries (4 items, range: 4–20), satisfaction (15 items, range: 15–75), and social worries (7 items, range: 7–35). The Es-DQoL score ranges between 43 (highest level of QoL) and 215 (lowest level of QoL). In order to facilitate data comprehension, the variable “Total EsDQoL score” and subscales scores were presented on a scale of 0–100, where the higher the score, the higher QoL, using the following formula: (Transformed score−minimum possible score)/(maximum possible score-minimum possible score) × 100, where “Transformed score” is calculated as maximum possible score−(real score-minimum possible score).

### Clinical evaluation

Clinical and sociodemographic variables were collected at study entry; biochemical variables (HbA_1c_ levels) were taken from medical records (most updated record within a 3-month period before study entry). Variables included age; gender; level of education; marital status; age at disease onset; disease duration; HbA_1c_ levels; presence of chronic diabetes complications; ketoacidosis episodes and/or other diabetes-related hospital admission (excluding hospital admission at onset); severe hypoglycemic episodes, and total hypoglycemic and hyperglycemic episodes in the previous 15 days. Glycemic-related variables were collected from self-monitoring glucose data (patient electronic records or glucose diary). Glycemic instability, severe hypoglycaemia, unawareness hypoglycaemia, chronic diabetes complications, retinopathy, nephropathy and evaluation of diabetic peripheral neuropathy and finally, peripheral vascular complications have been defined previously [19–21].

### Psychological evaluation

Psychological assessment was carried out at outpatient facilities and included a structured diagnostic interview by a psychologist included in the research team through *Mini International Neuropsychiatric Interview (MINI)* [22]. This planned interview aimed at detecting psychiatric disorders such as depressive and/or anxious symptoms. Self-reported questionnaires were used for grading severity of psychological symptomatology (Beck Depression Inventory II (BDI-II) [23], State-Trait Anxiety Inventory, Form Y (STAI-Y) [24, 25]), detecting Fear of hypoglycemia (FH), (Fear of hypoglycaemia Scale (FH-15), cut-off score 28 [26]) and determining the social support received by patients (Medical Outcomes Study (MOS) Social Support Survey [27, 28]) according to standard procedures.

### Statistical analysis

Sample size was calculated in order to detect a minimum difference of 4 points at Total Es-DQoL score between male and female subgroups, a 95% confidence level, a statistical power of 90% and assuming a 25% drop-out rate. The required estimated sample size was 420 patients. Normally distributed variables are presented as mean and standard deviation (SD). Variables with skewed distribution (Es-DQoL and subscales) are presented as geometric means and interquartile range. In order to detect gender differences, qualitative variables were compared using Chi squared test and quantitative variables using t-Student test. Pearson´s correlation coefficient (rho) was used in univariate analyses for quantitative variables. A Stepwise multiple linear regression was applied to explore predictors for QoL in our sample of patients. Variables included in this analysis were significant in univariate analyses, demographic, clinical and disease-specific variables, as well as the scores on psychological tests. For the analysis of the data, the statistical package package IBM SPSS Statistics 22.0 for Windows and statistical significance was defined as ≤ p 0.05.

## Results

A total of 464 adults were randomly selected, 312 of whom finished complete evaluation and were included in the analysis. Among 152 non-included adults, 72 (47.3%) had one or more exclusion criteria, 55 (36.2%) were not interested in the study and 25 (16.4%) could not come to the appointment because of difficulties in obtaining work permit or the impossibility to travel to the hospital. The non-enrolled population was similar to the enrolled population in age, gender, age at diagnosis and duration of diabetes (data not shown).

Sociodemographic, clinical variables and prevalence of depression, anxiety and fear of hypoglycemia of the patients enrolled in the study are summarized in Table 1. Social support was comparable between female and male subgroups (84.9 ± 12.0 vs. 83.1 ± 15.2, *p* ns).

**Table 1:** Sociodemographic and clinical data

	Total	Male	Female
Number of patients (n, %)	312, 100%	161, 51.6%	151, 48.4%
Age (years)	38.2 ± 12.7	38.7 ± 12.7	37.7 ± 12.6
Education (years)	13.4 ± 4.7	13.2 ± 4.8	13.6 ± 4.7
Marital status:			
Single	103 (33%)	54 (33.5%)	49 (32.4%)
Married	151 (48.4%)	75 (46.5%)	76 (50.3%)
Divorced	23 (7.3%)	15 (9.4%)	8 (5.4%)
Widowed	6 (1.9%)	2 (1.3%)	4 (2.6%)
In a relationship	29 (9.3%)	15 (9.3%)	14 (9.3%)
Age at diagnosis (years)	17.7 ± 10.7	17.6 ± 11.1	17.7 ± 10.3
Duration of diabetes (years)	20.4 ± 12	20.7 ± 12.4	19.9 ± 11.6
HbA_1c_ (%)	7.5 ± 1.1	7.5 ± 1.2	7.5 ± 1
Glycemic Instability^a^	9.2 ± 8.3	9.4 ± 9.3	8.4 ± 7.1
Hypertension	77 (24.6%)	47 (29.2%)	30 (19.9%)*
Unawareness hypoglycemic	95 (30.4%)	54 (33.5%)	41 (27.1%)
Severe hypoglycemia (last year):			
0	244 (78.2%)	127 (78.9%)	117 (77.5%)
1	35 (11.2%)	18 (11.2%)	17 (11.25%)
> 1	33 (10.6%)	16 (9.9%)	17 (11.25%)
Intensive insulin regimen:	312 (100%)	161 (100%)	151 (100%)
Bolus Basal	287 (92%)	152 (94.4%)	135 (89.4%)
CSII	23 (7.4%)	9 (5.6%)	14 (9.3%)
Insulin regimen < 2 shots a day	2 (0.6%)	0 (0.0%)	2 (1.3%)
Microvascular complications:	192 (61.5%)	108 (67%)	84 (55.6%)
Retinopathy	107 (34.3%)	61 (37.9%)	46 (30.5%)
Nephropathy	62 (19.9%)	34 (21.1%)	28 (18.5%)
Neuropathy	23 (7.4%)	13 (8.1%)	10 (6.6%)
Macrovascular complications	14 (4.5%)	11 (6.8%)	3 (1.9%)*
Fear of hypoglycemia	139 (44.6%)	70 (43.5%)	69 (45.7%)
Depression (MINI)	72 (23.1%)	24 (14.9%)	48 (31.7%)*
Anxiety (MINI)	56 (17.9%)	21 (13%)	35 (23.2%)*

Data are expressed as Mean ± SD or n (%)

Prevalence of Fear of Hypoglycemia is calculated according to FH-15 questionnaire

CSII, Continuous subcutaneous insulin infusion; MINI, Planned psychological interview

^a^Glycemic instability is defined as number of episodes of severe hypoglycaemia, mild hypoglycaemia (≤ 70 mg/dl) and hyperglycemic excursions (≥ 250 mg/dl) in 15 days prior to evaluation

*p < 0.05

### Quality of life evaluation

QoL assessed by Es-DQOL and its subscales, is described in Table 2. Compared to male patients, female patients showed lower QoL [75 (IC95% 73.6–77.5) vs. 80 (IC95% 75.7–83.1), p < 0.05] and scored significantly worse in subscale Diabetes-related worries [69 (IC95% 50.0–81.0) vs. 75 (IC95% 72.9–79.0), p < 0.05].

**Table 2:** Total QoL and subscales

	Total (n = 312)	Male (n = 161)	CI	Female (n = 151)	CI	p value
Satisfaction score (15 items)	75 (61–86)	77 (64–87)	74.8–81.6	73 (60–85)	72.0–78.3	0.180
Diabetes Impact score (17 items)	81 (74–90)	84 (75–90)	80.2–87.0	80 (72–89)	78.6–84.9	0.192
Social worries score (7 items)	78 (64–92)	82 (67–92)	79.5–84.1	78 (60–93)	76.2–82.0	0.226
Diabetes-related worries score (4 items)	75 (56–81)	75 (62–87)	72.9–79.0	69 (50–81)	66.0–71.3	0.005*
Total Es-DQoL score (43 items)	78 (68–86)	80 (69–87)	75.7–83.1	75 (65–85)	73.6–77.5	0.041*

Score range for Total Es-DQoL score and its subscales are 0–100: values are expressed by median and interquartile range; *p < 0.05. CI 95%-confidence interval

### Psychological evaluation

Among female patients, higher frequency of depression [31.7% (IC 95% 26.2–40.8) vs. 14.9% (IC 95% 10.1–20.8), p < 0.05] and anxiety [23.2% (IC 95% 19.3–33.14) vs. 13.0% (IC 95% 8.1–18.4), p < 0.05] was found; moreover, severity of depressive and anxious symptoms was higher in female subgroup (Table 3). No difference in prevalence of fear of hypoglycaemia [45.7% (IC95% 36–54) vs. 43.5% (IC95% 38–51), *p ns*] between DM1 female and male subgroups was observed.

**Table 3:** Score in psychological tests

Variables	Total (n = 312)	Male (n = 161)	CI	Female (n = 151)	CI
Fear of hypoglycemia	26 (20–33)	25 (19–32)	25.3–28.2	26 (21–34)	24.4–27.6
Depression (Beck)	4 (2–11)	4 (2–9)	3.0–5.1	5 (2–13*)	3.8–6.4
State anxiety (STAI-S)	13 (7–22)	12 (6–18.5)	10.3–13.7	15 (9–27*)	13.5–17.5
Trait anxiety (STAI-T)	16 (10–26)	15 (8.5–24.5)	14.1–17.4	18.5 (11–27*)	16.0–20.7

Values are expressed by median and interquartile range; *p < 0.05. CI 95%-confidence interval

### Predictive factors of quality of life

To study the association between QoL and demographic and clinical variables, univariate analyses were performed. The following demographic and clinical variables were included in the model: age, school education; age at diagnosis; duration of diabetes; HbA_1c_; glycemic instability; severe hypoglycemia (previous year); unawareness hypoglycemia; presence or absence of macrovascular and microvascular complications; presence of depression; presence of fear of hypoglycemia; presence of anxiety; severity of depressive and anxious symptoms according to scores in specific questionnaires. The presence of macrovascular complications, unawareness hypoglycemia, history of severe hypoglycemia and glycemic instability were associated with worse QoL in the univariate analysis (*p *< 0.05). Among psychological variables, the presence of depression, anxiety and fear of hypoglycemia were also significantly associated with worse QoL (*p *< 0.05). This association was shown both in non gender- and gender-specific analysis. As for psychological test scores, lower scores in MOS, that relates to inadequate social support, were also associated to worse QoL in non gender- and gender-specific analysis. Inversely, higher scores in the depression, anxiety and fear to hypoglycemia questionnaires were associated with worse QoL, both in non gender- and gender-specific analysis (*p *< 0.01).

In the multivariable analysis the variables that showed a significant association in the univariate analysis were included. The results show that severity of symptoms of depression, anxiety, fear of hypoglycemia, higher glycemic instability and the presence of macrovascular complications were predictive factors of QoL, with a R^2^ of 64.2% (Table 4). In a gender- specific analysis, severity of symptoms of depression and anxiety and fear of hypoglycemia were QoL predictors in male and female patients (Table 5), while glycemic instability was a female-specific QoL predictor (Table 6).

**Table 4:** Clinical and psychological predictive factors of QoL in multivariable regression model

	Beta coefficient	Standard error	t	p value	R^2^ adjusted
Depression (Beck score)	− 0.602	0.121	− 4.980	< 0.001	0.642
Fear of hypoglycemia (FH-15 score)	− 0.499	0.071	− 7.1	< 0.001	
Glycemic instability^a^	− 0.234	0.082	− 2.855	< 0.005	
Macrovascular complication (yes/no)	− 6.976	3.558	− 1.961	< 0.05	
Trait anxiety (STAI-T score)	− 0.288	0.076	− 3.775	< 0.001	

^a^Glycemic instability is defined as number of episodes of severe hypoglycaemia, mild hypoglycemia (≤ 70 mg/dl) and hyperglycemic excursions (≥ 250 mg/dl) in 15 days prior to evaluation

**Table 5:** Clinical and psychological predictive factors of QoL in multivariable regression model (Male patients)

	Beta coefficient	Standard error	t	p value	R^2^ adjusted
Depression (Beck score)	− 0.879	0.147	− 5.979	< 0.001	0.717
Fear of hypoglycemia (FH-15 score)	− 0.520	0.085	− 6.098	< 0.001	
Trait anxiety (STAI-T score)	− 0.275	0.088	− 3.112	< 0.005

**Table 6:** Clinical and psychological predictive factors of QoL in multivariable regression model (Female patients)

	Beta coefficient	Standard error	t	p value	R^2^ adjusted
Trait anxiety (STAI-T score)	− 0.415	0.130	− 3.207	< 0.005	0.544
Fear of hypoglycemia (FH-15 score)	− 0.540	0.113	− 4.797	< 0.001	
Depression (Beck score)	− 0.484	0.195	− 2.483	< 0.05	
Glycemic instability*	− 0.295	0.136	− 2.163	< 0.05	

*Glycemic instability is defined as number of episodes of severe hypoglycaemia, mild hypoglycemia (≤ 70 mg/dl) and hyperglycemic excursions (≥ 250 mg/dl) in 15 days prior to evaluation

## Discussion

In our study, DM1 female patients showed lower QoL and scored significantly worse in the Diabetes-related worries subscale. These findings are in agreement with published data that show that female gender is associated with poorer QoL, both in adults [10, 14] and adolescents [13, 29, 30]. When analysing QoL subscales, our results are also concordant with those published by Trento et al. that showed higher diabetes-related worries among female DM1 patients [13]. Sociodemographic profile, clinical variables and social support were similar between male and female subgroups and could not explain poorer QoL in our DM1 female cohort. Only higher prevalence of hypertension and lower prevalence of macrovascular complications among females were clinically relevant between subgroups. Moreover, use of continuous subcutaneous insulin infusion as intensive insulin therapy was higher among females, which could bias (if present) the result to better QoL scores [31]. Thus, these findings reinforce the robustness of lower female-associated QoL.

According to our data, severity of depressive and anxious symptoms emerged as factors independently associated to lower QoL in both male and female patients. In DM1, gender differences were previously described in psychological adjustment. The psychological factors that negatively relate with psychological adjustment in women compared to men are worse depressive coping and depressive symptomatology [32, 33]. Thus, decreased QoL and specifically higher diabetes-related worries could be explained by a higher prevalence of depression and anxiety and higher intensity of depressive and anxious symptoms in our female cohort.

As expected, fear of hypoglycaemia has a relevant psychological impact in DM1 adult population, and was independently associated to poor QoL in both male and female patients. Controversial data on gender differences in fear of hypoglycemia were previously published in DM1 diabetic adults and adolescents [34, 35]. Anderbro et al. reported higher fear of hypoglycemia among female patients. As a comprehensive psychological evaluation was lacking, authors could only hypothesize that a higher sensitivity to anxiety disorders in female adult population could partially explain this differences [34]. In our cohort, higher prevalence of fear of hypoglycemia among female patient was not found as previously described in adolescent female patients [35]. According to our data, unpredictable glycemic control and psychological impact of fear for hypoglycaemia in this context are predictive factors of low QoL, a clinical relevant finding as exposure to hypoglycaemia and its effect could be avoided and specifically managed.

Interestingly, glycemic instability emerged as a female-specific factor which adversely affected QoL. Published data are not consistent with the association of QoL and metabolic control, assessed by HbA_1c_ levels [11, 36–39], the standard method for assessing glycemic control. However, used as a sole marker of glycemic control, HbA_1c_ does not reflect intra- and interday glycemic excursions that may lead to hypoglycemia or hyperglycemia events. Hypoglycemic episodes in DM1 patients and worries about their consequences on personal, social, and professional areas negatively impact on QoL of patients and their families [40]; among DM1 children and adolescent patients, hyper- and hypoglycemic episodes were associated to low QoL [41]. Men and women have different attitudes and behaviors related to diabetes care [42] as women have a greater interest and concern for diabetes and are more likely to perceive symptoms [43]. Thus, gender-related differences in the effect of hyper- and hypoglycemic episodes on QoL is plausible. As far as we are concerned, an association between frequency of uncontrolled glycemic excursions and low QoL among female patients has not been previously described. A stricter follow up of hypo/hyperglycemic episodes in clinical practice, through interstitial glucose monitoring, such as flash glucose monitoring or continuous glucose monitoring, could reduce their frequency, thus focussing interventions beyond HbA_1c_ aims and improving QoL of patients with DM1 [44, 45].

Our study has limitations as a deep sociodemographic evaluation was not performed and relevant variables related to health-related QoL, such as occupational status or level of employment, are lacking [14]. Even though the large cohort included only DM1 adult population followed in a tertiary care setting. On the other hand, a complete psychological and clinical evaluation performed allowed to identify clinically relevant conditions; those related to glycemic control would not had been detected through a classical HbA_1c_-centered evaluation. Intervention studies are needed to specifically test the hypothesis of improvement of QoL through reduction of hypo- and hyperglycemic excursions in this clinical setting.

## Conclusions

This study shows lower QoL among adult women with DM1 and identifies modifiable, QoL-associated variables. Higher severity of depressive and anxious symptoms and a more negative impact of glycemic excursions in female patients could partially explain lower QoL. This study could help health care providers to identify patients at higher risk of lower QoL and reinforce the relevance of educational, technological and psychological interventions aimed at reducing glycemic variability and improving psychological status to increase QoL in DM1 patients.

## Data Availability

The datasets used and/or analysed during the current study are available from the corresponding author on reasonable request.

## Abbreviations

QoL: Quality of life
DM1: Type 1 diabetes mellitus
DQoL: Self-administered Diabetes QoL questionnaire
Es-DQoL: Self-administered Diabetes QoL questionnaire in Spanish
UHVR: University Hospital Virgen del Rocío (UHVR)
ADA: American Diabetes Association
FH-15: Fear of hypoglycaemia Scale
BDI-II: Beck Depression Inventory II
STAI-Y: State-Trait Anxiety Inventory, Form Y
MOS: Medical Outcomes Study Social Support Survey
SD: Standard deviation
HbA_1c_: Glycated hemoglobin
